# Can delivery mode influence future ovarian reserve? Anti-Mullerian hormone levels and antral follicle count following cesarean section: a prospective cohort study

**DOI:** 10.1186/s13048-019-0551-z

**Published:** 2019-09-03

**Authors:** Ashraf Moini, Reihaneh Pirjani, Maryam Rabiei, Maryam Nurzadeh, Mahdi Sepidarkish, Reihaneh Hosseini, Ladan Hosseini

**Affiliations:** 1 Department of Endocrinology and Female Infertility, Reproductive Biomedicine Research Center, Royan Institute for Reproductive Biomedicine, ACECR, Tehran, Iran; 2 Obstetrics and Gynecology Department, Arash Women’s Hospital, Tehran University of Medical Sciences, Tehran, Postal code: 1653915911 Iran; 3 Department of Biostatistics and Epidemiology, Babol University of Medical Sciences, Babol, Iran; 4 Research development center, Arash Women’s Hospital, Tehran University of Medical Sciences, Tehran, Iran

**Keywords:** Anti-Mullerian hormone, Antral follicle count, Caesarean section, Delivery mode, Ovarian reserve

## Abstract

**Background:**

The incidence of Cesarean has increased in recent years. The purpose of this study is to evaluate the effect of cesarean section on ovarian reserve*.*

This is a prospective cohort study from January 2016 to November 2017. Inclusion criteria included singleton primigravid pregnant women whose gestational age was above 37 weeks. Exclusion criteria included history of infertility, pelvic surgery, underlying chronic diseases, any adverse pregnancy outcome and postpartum complication in current pregnancy and hormonal medication within six months of delivery. Anti-Mullerian hormone was measured at the admission time for delivery. The type of delivery was determined based on obstetrics indications. Six months after delivery, antral follicle count was performed and anti-Mullerian hormone was measured again.

**Result(s):**

First blood sample was taken from 730 women. After excluding 550 women, the second blood sample was taken from 180 participants. The mean of first anti-Mullerian hormone in women with cesarean and vaginal delivery were 1.01 ng/mL (95% CI 0.82 to 1.18) and 1.18 ng/mL (95% CI 0.96 to 1.40) respectively (*P* = 0.211). The mean of second anti-Mullerian hormone in women with cesarean and vaginal delivery were 4.77 ng/mL (95% CI:3.91 to 5.63) and 4.92 ng/mL (95% CI: 4.01 to 5.82) respectively (*P* = 0.818). No statistically significant difference existed in total AFC between cesarean and vaginal delivery groups (MD: 0.41, 95% CI: − 1.05 to 1.89, *P* = 0.576).

**Conclusion:**

Antral follicle count and anti-Mullerian hormone, six month after delivery, are not affected by delivery mode even after adjusting for women’s age, baseline Anti-Mullerian hormone, body mass index, gestational age at delivery, breastfeeding, postpartum menstruation, neonatal sex and weight. Based on our best knowledge, this is the first report that investigates the effects of delivery mode on ovarian reserve. Decreased fertility following cesarean has been shown in some previous studies but most of them had assessed this association based on the incidence of subsequent pregnancy. Since subsequent pregnancy can be influenced by several confounding factors, we investigated the effect of cesarean on fertility using its impact on anti-Mullerian hormone levels and antral follicle count. We hope that this study will be a beginning of more detailed studies in this field. We believe that this link is yet to be studied.

## Introduction

Cesarean Section (CS) is one of the most common pelvic surgeries. The incidence of CS has increased in recent years [1–4]. Over the recent years, women have tended to become pregnant at older ages. Concerns about long-term negative effects of CS on subsequent fertility are increasing due to growing prevalence of CS and rising age at first pregnancy. Certainly, by postponing the first pregnancy, the importance of reducing the rate of ovarian reserve loss over the next years will be highlighted, so that the chance of future pregnancies will decrease.

Over the past years, several studies have been conducted to determine the effect of CS on future fertility. [1–6] and most of them have shown a negative relationship between CS and future fertility [4–7]. Subsequent pregnancies and births can be affected by several factors. Maternal parity, indication of previous CS such as twin pregnancy, history of previous treatment for infertility, intrapartum and postpartum experiences, and even maternal desire for future pregnancy and many other factors can affect the results. Given the above, it is essential to evaluate the effect of CS on fertility with more care.

Anti-Mullerian hormone (AMH) level is correspondant with the number of antral follicles and its level decreases with increasing age and decreasing the number of antral follicles [8]. AMH levels are constant across the menstrual cycle and its serum levels are not affected by FSH, LH, and E2 levels [8]. These unique characteristics of AMH have made it a good predictor of ovarian reserve. In addition to AMH, the antral follicle count (AFC) on the third day of the menstrual cycle is also a good marker for ovarian reserve evaluation [9, 10].

Since CS is one of the most common surgeries on the uterus and also preserving ovarian reserve after the first pregnancy is crucial for achieving the next successful pregnancy, we have decided to examine the effect of CS in comparison with vaginal delivery (VD) on the serum levels of AMH and AFC as markers of ovarian reserve because these two can predict the future fertility.

## Material and methods

This prospective cohort study was conducted at a university hospital (Arash hospital, affiliated to Tehran University of Medical Sciences) from January 2016 to November 2017. All singleton pregnant women who attended the hospital for delivery were invited to study based on inclusion and exclusion criteria. Inclusion criteria included singleton primigravid pregnant women who were 18–45 years old and had conceived spontaneously and their gestational age was above 37 weeks of pregnancy. Exclusion criteria included history of previous pregnancy and abortion, history of infertility, pelvic surgery, autoimmune diseases, chemotherapy, radiotherapy, underlying chronic diseases, adverse pregnancy outcomes including gestational diabetes, preeclampsia, gestational hypertension, preterm labor and fetal intrauterine growth restriction, any postpartum complication including postpartum hemorrhage, blood transfusion, postpartum metritis, postpartum fever and infection from any source, any kind of incision on the uterus other than kerr incision, hormonal drugs or contraceptives consumption within six months of delivery. After the consent was signed, their demographic information was collected through a questionnaire and also by using their case records. The venous blood samples were collected from the participants at the time of admission for delivery. Blood samples were frozen and stored at − 70 °C until they were analyzed. The type of delivery (CS or VD) was determined based on obstetrics indications.

Six months after delivery (on the third day of the menstrual cycle with the exception of those who experienced breastfeeding amenorrhea), during the next visit, participants completed a questionnaire including duration of breastfeeding, contraception method, menstrual cycle, and medical and drug history. If the participants used hormonal drugs and contraceptives six months after delivery, the second sample would not be taken and they were excluded from the study. A second blood sample was then obtained from the remaining participants and the same day vaginal sonography for evaluating AFC was performed on them. Serum AMH was measured by enzyme-linked immunosorbent assay (ELISA) using the AMH Gen II (Beckman Coulter assay).

Ultrasound examinations were performed with a C9-4V-MHz transvaginal probe (fillips affinity 50) to determine the number of antral follicles. The ovaries were evaluated in their transverse and longitudinal planes and the antral follicles were counted and measured. Antral follicles defined as follicles measuring 2 to 10 mm in diameter. The size of the follicle was considered as the mean of two perpendicular diameters, one of which should be the largest dimension of each follicle [11, 12]. All these measurements were carried out by two people (R.P and M.R) in order to avoid any error. Less than 4 antral follicles were defined as low AFC which suggests poor ovarian reserve. Ultrasound examinations were carried out on the third day of the menstrual cycle with the exception of those who experienced breastfeeding amenorrhea that had undergone ultrasound examination each day of the sixth month of delivery.

All statistical analyses were performed with Stata software for Windows, release 12.0 (Stata corporation, College Station, TX, USA). Baseline characteristics were compared among the women with CS and VD using the Independent-samples t-test for continuous data and a chi-square test for categorical data. Multiple Linear Regression was used to examine the association between delivery type and study outcomes (AMH and AFC) controlling for potential confounders. Potential confounders were women’s age, baseline AMH, body mass index (BMI), gestational age at delivery, breastfeeding, postpartum menstruation, neonate sex and weight. The magnitude of the effect is presented as coefficient regression and its 95% confidence interval.

### Ethical approval

This study was approved by the Institutional Review Board and the Ethics Committee of Tehran University of Medical Sciences, Tehran, Iran. (IR.TUMS.MEDICINE.REC.13964207) and all participants submitted written informed consent.

## Results

**Fig. 1:**
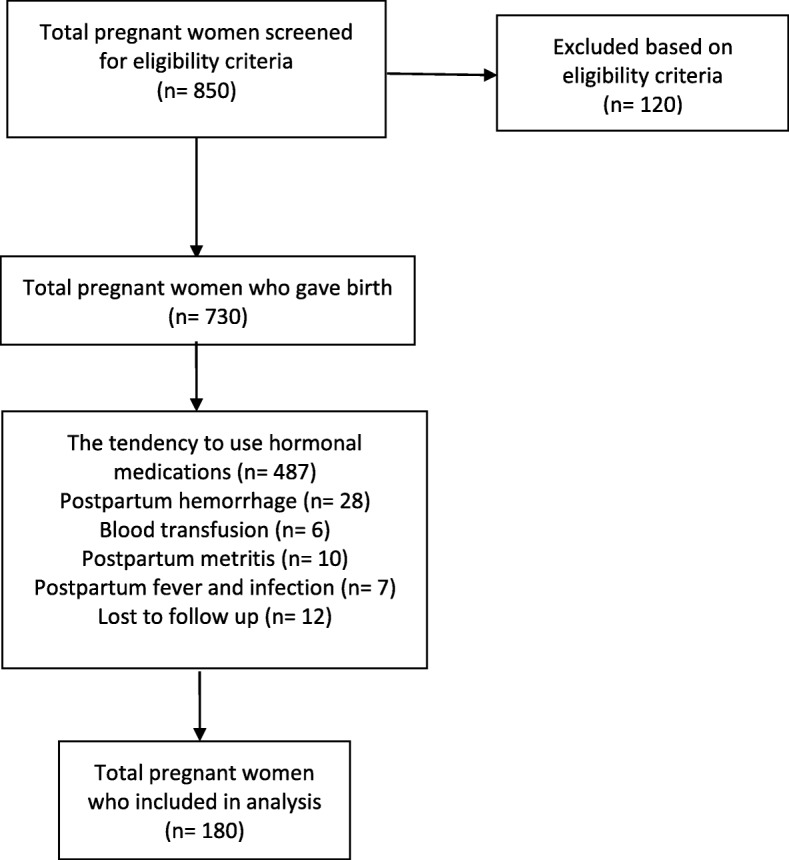
Flow diagram of pregnant women recruitment

Between January 2016 and September 2017, 850 pregnant women were assessed for eligibility criteria. Hundred and twenty women were excluded from the study due to one of the following causes: previous pelvic surgery, underlying chronic diseases, previous abortion, history of infertility, gestational diabetes, preeclampsia, gestational hypertension, preterm labor and fetal intrauterine growth restriction. After excluding them, 730 were remained in the study and the first blood sample was taken from them. It should be noted that at the time admission time, a large number of participants had not yet decided on their postpartum contraception method, so the first blood sample was taken from them. Among whom, 487 pregnant women tended to use hormonal medications within six months of delivery and 51 women experienced one of the following postpartum complications: postpartum hemorrhage, blood transfusion, postpartum metritis, postpartum fever and infection. So, 538 women were excluded after delivery too. Of 192 remaining pregnant women, 12 of them were lost the follow-up and did not return six months after delivery and finally the second blood sample was taken from 180 participants. On the same day that second blood samples were taken, ovarian antral follicles were also examined by sonography. Eventually survey was conducted on these 180 women. They were divided into two groups according to their delivery type. 95 women (52.8%) were classified in CS group, and the remaining 85 (47.2%) women were classified in VD group (Fig. 1).

**Fig. 2:**
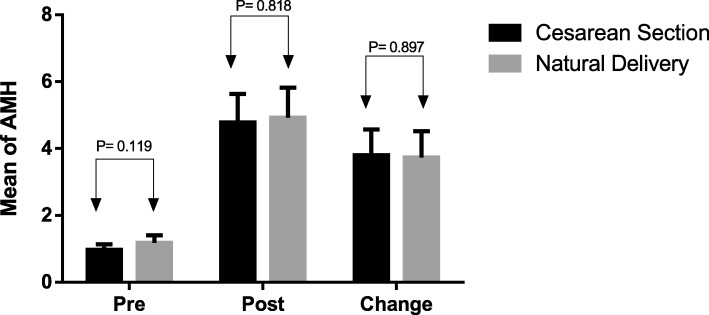
The mean of AMH at the beginning of study and after six months between cesarean section and natural vaginal delivery

Table 1 summarizes patients’ demographic characteristics, baseline AMH, and AFC in the two study groups. There was no difference between two groups regarding age, BMI, breastfeeding, postpartum menstruation and total number of AFC in the right and left ovaries. The mean AMH levels in women with CS were 1.01 ng/mL (95% CI 0.82 to 1.18), and in women with VD were 1.18 ng/mL (95% CI 0.96 to 1.40) at the beginning of the study (MD: -0.17, 95% CI: − 0.45 to 0.10, *P* = 0.211). Six months after childbirth, the mean AMH levels in women with CS were 4.77 ng/mL (95% CI:3.91 to 5.63), and in women with VD were 4.92 ng/mL (95% CI:4.01 to 5.82) (MD: -0.14, 95% CI: − 1.38 to 1.09, *P* = 0.818). Also there was no difference between two groups regarding the change of AMH (MD: -0.07, 95% CI: − 1.01 to 1.15, *P* = 0.897) (Fig. 2). After adjustment for women’s age, baseline AMH, BMI, gestational age at delivery, breastfeeding, postpartum menstruation, neonatal sex and neonatal weight, using a Linear Regression Model, there was no association between the delivery type (standardized coefficient: 0.74, 95% CI:-0.24 to 1.73, *P* = 0.137) and the AMH levels.

**Table 1:** Patients’ demographics, baseline AMH, and AFC

Variable	Women with CS (*n* = 95)	Women with VD (*n* = 85)	*P*-value
Age	27.42 (5.42)	26.20 (4.76)	0.112
BMI	24.03 (4.20)	24.03 (3.99)	0.997
Gestational age at delivery	39.03 (3.90)	38.89 (1.07)	0.997
Neonate weight	3296.33 (407.02)	3173.33 (487.35)	0.067
AFC (left ovary)	5.66 (2.90)	5.52 (2.90)	0.754
AFC (right ovary)	5.83 (2.66)	5.55 (2.42)	0.471
AMH^1^	1.01 (0.88)	1.18 (1.01)	0.211
AMH^2^	4.77 (4.20)	4.92 (4.19)	0.818
AMH^2^_−_ AMH^1^	3.80 (3.74)	3.73 (3.62)	0.897
Neonate Sex			
Male	44 (46.3)	52 (38.8)	0.046
Female	51 (53.7)	33 (61.2)	
Breastfeeding			
Yes	69 (72.6)	63 (74.1)	0.822
No	26 (27.4)	22 (25.9)	
Postpartum menstruation			
Yes	68 (71.6)	57 (67.1)	0.511
No	27 (28.4)	28 (32.9)	

^1^At the beginning of study

^2^After six months

No statistically significant difference existed in total AFC between the CS (11.50, 95% CI:10.42 to 12.57) and VD (11.08, 95% CI:10.07 to 12.08) groups (MD: 0.41, 95% CI: − 1.05 to 1.89, *P* = 0.576). We examined total AFC as a continuous variable in a Linear Regression Model, with very similar results. There was no association between delivery type (standardized coefficient: 0.69, 95% CI:-0.83 to 2.22, *P* = 0.372) and total AFC even after adjustment for confounding factors, including women’s age, baseline AMH, BMI, contraceptive method, gestational age at delivery, breastfeeding, postpartum menstruation, neonatal sex and neonatal weight.

## Discussion

Based on our best knowledge, this is the first report that investigates the effects of delivery mode on ovarian reserve. The objective of this study was to evaluate the effect of CS in comparison with VD on ovarian reserve. In the present study, ovarian reserve has been assessed by means of maternal serum AMH and AFC. The results showed that delivery mode had no effect on ovarian reserve after adjusting for women’s age, baseline AMH, BMI, gestational age at delivery, breastfeeding, postpartum menstruation, neonatal sex and neonatal weight.

Over the recent years, several observational studies have examined the relationship between CS and future fertility and some of them concluded that the prevalence of subsequent pregnancy following CS declined [3–7]. Nevertheless, some other studies have not seen such a relationship [1, 3]. Two systematic review and meta-analysis have been conducted on the effect of CS on future fertility and pregnancy [4, 7]. In one of them, it was concluded that CS might have a negative impact on future pregnancies but nonexperimental survey data and selection bias in its included studies were likely to affect the results [7]. As the previous study, the results of the other systematic review showed that CS is correlated with decreased subsequent fertility in comparison with VD [4]. But according to characteristics of included studies in this review article that some of them had not had risk adjustment, we think that the results should be interpreted with caution. The authors also stated that even if there is an impact of CS on subsequent pregnancy, this effect is meager [4]. As it is clear from the above, most previous studies that examined the effect of CS on fertility have assessed this effect based on the incidence of subsequent pregnancy. Since subsequent pregnancy can be influenced by several confounding factors, we have investigated the effect of CS on fertility using its impact on ovarian reserve.

Engin Oral and KorayElter concluded that the effect of CS on future fertility is most likely due to some other confounding factors, rather than due to surgical impact of CS [13].

Many studies have also been carried out done about the effects of various types of pelvic surgery on ovarian reserve [14–16] and some of them have demonstrated that pelvic surgery may affect ovarian blood supply [14, 16]. Although we did not find any relationship between the CS and ovarian reserve, during CS, as with any other surgery, there is a potential risk of pelvic organs and vessels trauma due to pelvic manipulations and inflammatory mediators releases, which may lead to decreasing ovarian reserve.

Over the course of recent years, some studies have been conducted to evaluate AMH during pregnancy and postpartum [17–21], and it has been revealed that pregnancy has significant effect on serum AMH level in such a way that AMH level decreases progressively by advancing gestational age and maximum effect has been found in third trimester [19–21] Also in our study, the levels of serum AMH at the end of third trimester were lower than six months after delivery. On the other hand, in recent years, researchers have considered the relationship between serum AMH and adverse pregnancy outcomes [22–24]. Considering that there is still no consensus on the impact of pregnancy outcomes on the level of AMH and ovarian reserve and also our objective was to examine the effect of delivery type on the ovarian reserve, we tried to eliminate any potential factor that had effect on AMH. Therefore, all women who had any adverse pregnancy outcomes and postpartum complications were excluded from the study.

It is well-known that ovarian reserve decrease with increasing age. Although it may seem that a period of 6 months may be too short to judge about future fertility, many other factors might affect ovarian reserve over the course of time other than delivery mode. So, we decided to evaluate ovarian reserve no later than 6 months after delivery. We could not prove any association between CS and ovarian reserve but CS may cause subsequent infertility and subfertility through different mechanisms. Effect on the endometrial cavity and ensuing implantation and placentation, pelvic adhesion, and the possibility of affecting the pelvic organs blood supply, all can be considered as contributing factors in reducing fertility following CS. Also, uterine manipulation during CS and potential complications such as bleeding and infection may have a negative impact on ovarian reserve, so the results of our study should be interpreted with caution and much more studies are needed about this subject. We believe that association between delivery mode and ovarian reserve is yet to be studied.

One of the strengths of our study is that there was the least level of heterogeneity among the participants. Only singleton pregnancies were examined and participants were limited to women who had not had previous pregnancies and none of them had had underlying disease. There is controversy over the effect of smoking on AMH levels. Some studies have reported a decrease in blood hormone levels following smoking [25, 26], while others have shown no effect [27–29]. Given the uncertainty about the impact of smoking on serum AMH levels, we did not include smoker women in this study. On the other hand, due to the effect of contraceptive pills on the level of AMH and AFC, participants who had consumed various hormonal medications were excluded from the study. With reference to the above explanation, we tried to eliminate the confounding factors as much as possible, so that the effect of CS on ovarian reserve has been more accurately examined. Of course, there were some inevitable factors that might affect AMH concentration and AFC. One of these factors was breastfeeding that whose relationship with AMH concentration and ovarian reserve has not been identified in previous studies yet. The other inevitable factor is fetal sex. It is prominent that AMH blood concentration is higher in male fetuses. So, most probably fetal sex can affect the AMH concentration in maternal serum, as it has been observed in a study that serum AMH levels are significantly higher in mothers who have had a male fetus [30]. Considering the possible impact of these factors on the study outcomes, we analyzed their effect and it was demonstrated that the relationship between delivery mode and ovarian reserve was not affected by breastfeeding and the fetal sex.

One of the limitations of our study is that we did not differentiate between emergency and elective cesareans and also did not consider cervical dilatation and effacement during emergency cesarean section. However, since in this study CS has not had an effect on ovarian reserve, it does not seem that this limitation has a significant effect on the results. Another limitation of our study is AFC measurement timing. It is well-known that the best time to measure AFC is the third day of the menstrual cycle. However, a retrospective cohort study showed that AFC retains its predictive value when measured at different phases of the menstrual cycle [9]. In our study, some women experienced amenorrhea due to breastfeeding. Therefore, in this group of women, we had to measure AFC regardless of the menstrual cycle. However, using the Multiple Linear Regression Model, there was no difference in the AFC based on the existence of amenorrhea or not.

## Conclusion

The results showed that AFC and AMH concentration, six month after delivery, are not affected by delivery mode even after adjustment done for women’s age, baseline AMH, BMI, gestational age at delivery, breastfeeding, postpartum menstruation, neonatal sex and weight. Since the relationship between ovarian reserve and CS has not been sufficiently considered in previous studies and with the assumption that pelvic vessel manipulation during pelvic surgeries including CS may affect ovarian blood supply and also potential pelvic adhesions after CS may have impact on the ovarian reserve, we suggest that further studies be done on this issue. Since according to our best knowledge, this is the first study about delivery mode effect on ovarian reserve, we hope that this study will be a beginning of more detailed studies in this field.

### Twee table abstract

Anti-Mullerian hormone and Antral follicle count are not affected by delivery mode. That this link is yet to be studied.

## Data Availability

The datasets used and/or analysed during this study are available from the corresponding author on reasonable request.

## Abbreviations

AFC: Antral follicle count
AMH: Anti-Mullerian hormone
CS: Cesarean Section
VD: Vaginal delivery
